# Progress and Bottlenecks in Large-Scale Commercial Space Transportation Systems

**DOI:** 10.34133/research.1349

**Published:** 2026-07-03

**Authors:** Xiaowei Wang, Zhengyu Song, Weimin Bao

**Affiliations:** ^1^ China Academy of Launch Vehicle Technology, Beijing, China.; ^2^ China Association of Science and Technology, Beijing, China.

## Abstract

With global space activities shifting from an exploration-oriented focus to large-scale development and utilization, low-cost, “airline-mode”, and high-capability space transportation capabilities have become the critical foundation for supporting the construction of space infrastructure and the flourishing of the space economy. This paper targets the “dual-hundredfold” goal, aiming for a hundredfold increase in both payload insertion mass and launch frequency compared to current levels. After analyzing the development trends and challenges of large-scale space transportation systems, key technologies—such as ultra-high-speed re-entry active thermal management, long life-cycle engines, efficient low-temperature propellant transfer control and precise sensing, aerodynamic-assisted orbital maneuvers, aerodynamic and control integration design for the payloads featuring high lift-to-drag ratios and high packing densities, and fully electric-driven pressurization and fluid transport control technologies—are discussed. Breakthroughs in these technologies are expected to revolutionize human access to space, give rise to new intercontinental travel paradigms, and reshape the global technological competition landscape and strategic balance.

## Introduction

Space transportation constitutes the foundational infrastructure of all space activities [1–3]. Over the past several decades, China has independently developed the Long March series of launch vehicles through technological accumulation and innovation, building a comprehensive space launch capability from the ground up [4–6]. This capability has provided essential support for major space programs, including human spaceflight, the BeiDou navigation system, deep-space exploration, and satellite internet constellations.

Space engineering is inherently a complex, large-scale system, characterized since its inception by strong multidisciplinary coupling and system-level interactions. It integrates knowledge across physics, chemistry, mechanics, materials science, control engineering, electronics, and other disciplines. Successful implementation of space systems therefore requires the breaking down of disciplinary boundaries and deep interdisciplinary integration [7–9]. In particular, the space systems engineering theory pioneered by Qian Xuesen emphasizes a holistic and integrative perspective [10]. It begins with overall mission objectives, coordinates subsystem development, and targets comprehensive system integration. This methodology provides a key foundation for multidisciplinary collaboration in space engineering.

At present, global space activities are experiencing a period of rapid growth, with increasingly ambitious missions such as mega-constellation construction, lunar stations, and crewed Mars exploration. These missions demand that space transportation systems evolve beyond traditional architectures designed for single or limited mission types, toward capabilities that support the large-scale development and utilization of space. In such future scenarios, the required mass delivered to orbit may increase by 1 to 2 orders of magnitude compared with current levels, forming the basis for genuine industrial-scale space activities.

It is estimated that the demand for annual space access throughput will exceed 100,000 tons by the middle of this century [11–15]. Taking a standard single-launch orbital payload capacity of 10 to 20 tons as the baseline, the required annual launch frequency will reach 5,000 to 10,000 missions. At present, the global annual launch volume is only about 300 times, with a total orbital mass of approximately 3,000 tons. The current launch cost of mainstream launch vehicles ranges from 1,500 to 10,000 US dollars per kilogram to orbit. Accordingly, it is necessary to pursue a “double hundredfold” target, namely, achieving a hundredfold increase in both orbital payload mass and launch frequency relative to the current level. To fulfill this mid-century “double hundredfold” goal, a “double tenfold” development target is expected to be achieved around 2035.

Such a transition cannot be achieved through incremental upgrades alone. It introduces fundamentally new challenges that differ substantially from those addressed by conventional technological pathways. If these challenges are not addressed, space economy will face critical bottlenecks and its growth will be severely constrained.

Breaking through these limitations requires deeper interdisciplinary integration and increased emphasis on original innovations, thereby enabling the high-quality development of future space transportation systems. Against this backdrop, this paper systematically examines the development trends and key challenges associated with large-scale commercial space transportation systems, and provides a structured review of the core technological issues that must be addressed to enable scalable, sustainable space activities.

## Development Trends of Space Transportation Systems

### Large payload capacity, low cost, and high launch cadence as the dominant trends

With the accelerated exploitation of near-Earth space, global space activities are undergoing a transition from a mission-driven paradigm to a scale-oriented operational paradigm, imposing evolving demands on space transportation systems for larger payload capacity, lower cost, and higher launch frequency.

**Fig. 1.**
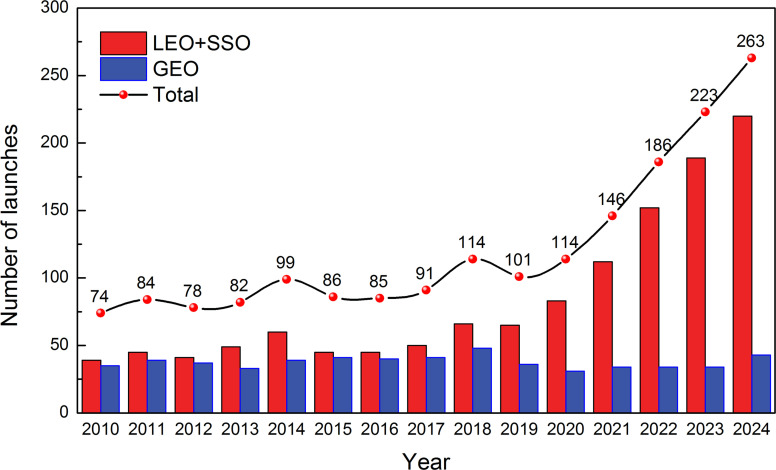
Annual number of orbital launches worldwide.

Figure 1 illustrates the deployment requirements of low Earth orbit (LEO) mega-constellations [16,17]. Globally, launches have exhibited an exponential growth trend over the past decade. These constellations may each comprise tens of thousands of satellites [18], creating a demand for launch systems capable of batch deployment, standardized interfaces, and high-density launch operations—requirements that far exceed the capabilities of existing launch systems. Constellations such as SpaceX Starlink, China SatNet, and Yuanxin are planned to deploy 42,000, 13,000 and 15,000 satellites, respectively, with full deployment scheduled to be completed by 2030 and 2035. The current launch capacity, launch frequency, and cost of the SpaceX Falcon 9 rocket and China’s launch vehicles are difficult to adapt to such deployment schedules. In particular, the mass of Starlink satellites keeps increasing, and more new constellation plans are continuously being proposed.

In the meantime, crewed lunar exploration initiatives such as the U.S. Artemis program [19–21] and China’s crewed lunar missions [22], as well as cislunar infrastructure construction and deep-space exploration activities are progressing steadily [23–26]. China has achieved lunar sample return [27,28] and will subsequently carry out Mars sample return missions [29]. These missions impose demanding requirements not only on payload capacity but also on system reliability, mission adaptability, and long-term operational sustainability.

Furthermore, emerging space activities, such as space tourism, on-orbit servicing and maintenance, and space resource utilization, are developing rapidly. Major space nations are actively promoting the concept of a cislunar econosphere, and space transportation is progressively exhibiting “airline-mode”, market-driven operational characteristics [30–32]. As stated in the Introduction, over the next 20 years, the demand for space access throughput will increase by 2 orders of magnitude compared with the present, and the existing launch capacity can no longer meet the deployment needs of current satellite constellations.

### Rapid advancement toward full reusable launch vehicles

Reusability represents a critical pathway for launch vehicles to return to the fundamental essence of space transportation systems. SpaceX has successfully achieved routine recovery and reuse of the Falcon 9 first stage [33–35], supporting the rapid deployment of the Starlink mega-constellation and establishing a pronounced first-mover advantage in LEO. As of December 2025, individual Falcon 9 first stages have been reused up to 32 times. The reuse turnaround cycle is approximately 9 d, demonstrating both the engineering feasibility and economic viability of “airline-mode” reuse. It has reduced the per-kilogram cost-to-orbit of mainstream U.S. launch vehicles from US$10,000 to approximately US$2,000 through reusability.

Nevertheless, the first stage accounts for 60% to 70% of the total launch vehicle cost, and its reuse can only reduce costs within this proportion. Therefore, to cut launch costs by an order of magnitude, full reusability of the launch vehicle is indispensable [36,37].

Building on this foundation, fully reusable launch vehicles will become the next hotspot, and the Super Heavy–Starship system is the pioneer among these. It is currently undergoing rapid iterative flight testing, targeting hundred-ton-class payloads to orbit, rapid turnaround, and extremely low marginal launch costs once operationalized, and is expected to confer generational advantages in LEO satellite launch, large-scale constellation deployment, and deep-space transportation.

From a global perspective, reusable launch technology has progressed from proof-of-concept exploration to a phase of system-level competition, and full reusability is the final target. Failure to achieve breakthroughs in this field may lead to a gradual loss of cost and scale advantages in future competition for space resources.

### On-orbit refueling enables medium- and high-orbit resource utilization

On-orbit refueling is being regarded as the next transformative technology following first-stage recovery, with the potential to fundamentally reshape space transportation architectures [38–41].

For missions involving medium and high Earth orbits, cislunar transfer, and deep-space exploration, traditional mission profiles—characterized by single ground-based fueling followed by a single launch to the target orbit—are constrained by ultimate launch capability, resulting in low efficiency and poor economic performance for large-scale medium- and high-orbit satellite markets. By enabling on-orbit refueling, ground launch and on-orbit transfer missions can be decoupled: Space vehicles may enter orbit with only their dry mass, followed by propellant replenishment tailored to mission requirements. This approach substantially improves payload fraction and operational flexibility.

Taking the LM-10 launch vehicle as an example, it sends a dry space transfer vehicle of about 70 t without propellant to LEO using its whole payload capacity, followed by on-orbit refueling of the vehicle. In this scenario, the departure mass of the transfer vehicle from LEO can be scaled up to approximately 700 t with more than 630 t of propellant, as summarized in Table 1. Such a mass is hardly achievable via a single launch by any heavy launch vehicle, thereby dramatically enhancing on-orbit transportation capability. Furthermore, the space transfer vehicle should be capable of long-term on-orbit reuse, which significantly reduces the cost of space transportation.

**Table 1.** Payload capacity comparison for different mission scenarios under 2 space transportation architectures

Transportation architecture	On-orbit departure mass (t)	Payload capacity (t)		
		Translunar injection (TLI)	LEO–HEO round trip	Earth–Moon round trip
Single-launch architecture of CZ-10 rocket	70	27	1.8	2.5
On-orbit-refueling-based architecture	700	278	36.4	46.3

**Fig. 2.**
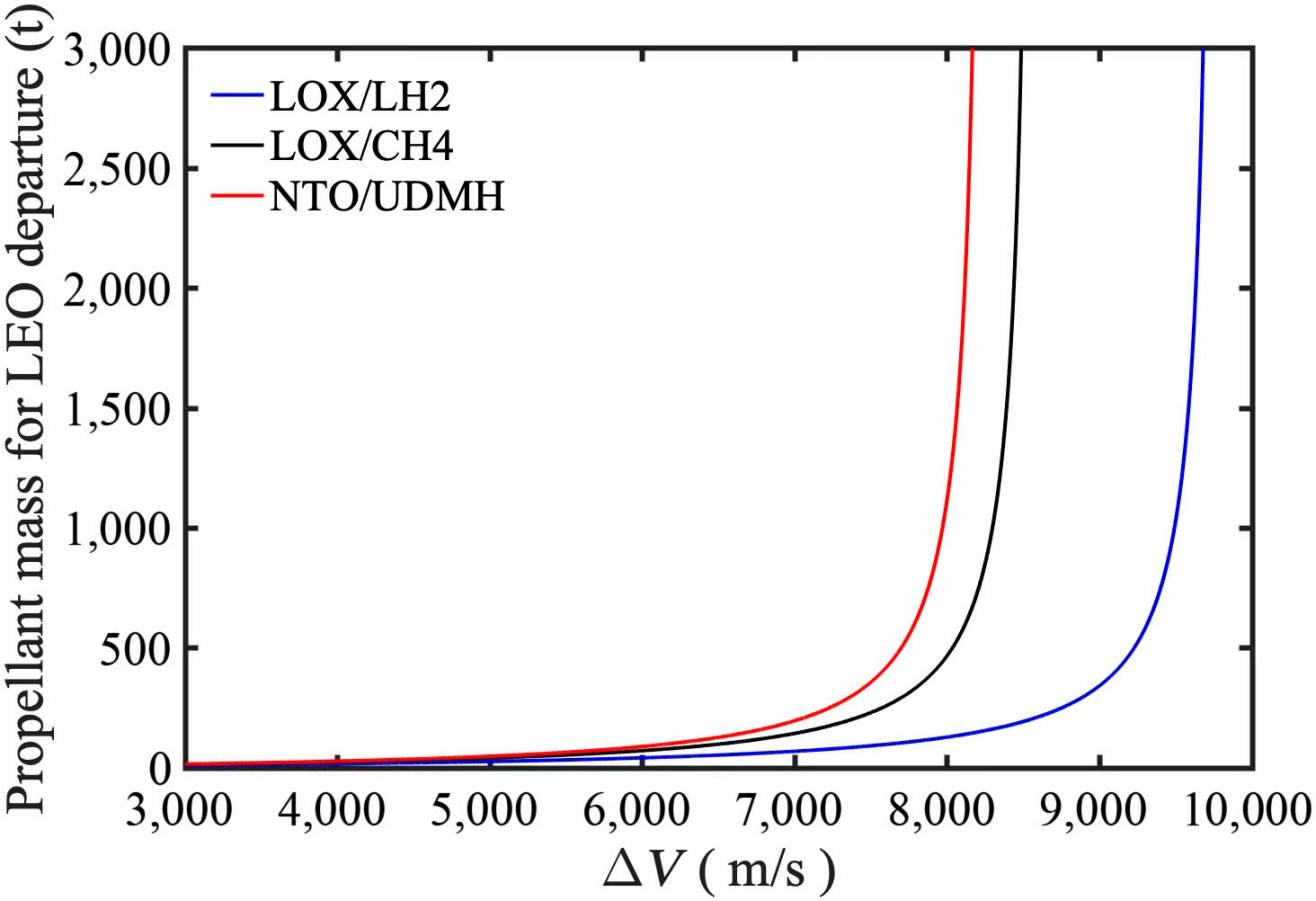
Comparison of required on-orbit departure mass using different propellant combinations.

Based on the Tsiolkovsky rocket equation, under the same requirements of payload mass and velocity increment, the scale of the launch vehicle has a nonlinear inverse relationship with the specific impulse of the propellant, as shown in Eq. (1). From the perspective of propulsion performance, for a given space transfer mission with fixed velocity increment requirements, the use of cryogenic propellants—such as liquid hydrogen/liquid oxygen (specific impulse up to 460 s) or liquid oxygen/methane (specific impulse up to 380 s)—offers significant advantages. This approach can reduce the required on-orbit departure mass substantially. This, in turn, leads to a comparable increase in payload capacity when compared with other storable propellants. The relationship between these factors is shown in Fig. 2.

$$\Delta v=v_{e}ln\frac{m_{0}}{m_{k}}$$ (1)

Table 2 presents a comparative analysis of on-orbit refueling requirements for different propellants under representative mission scenarios [42]. For example, to transport 10 t of effective payload for a round-trip mission between LEO and MEO (medium Earth orbit), the refueling mass required when using conventional propellants is 5 to 10 times greater than that required for cryogenic propellants.

**Table 2.** On-orbit refueling mass requirements for different propellant options under representative missions

Representative mission	Δv requirement (m/s)	Propellant	Specific impulse (s)	Refueling mass (t)
LEO–MEO round trip (10 t effective payload)	7,250	NTO/UDMH	350	258
		LOX/LCH_4_	380	179
		LOX/LH_2_	470	72

However, despite the numerous advantages of cryogenic propellants, their on-orbit storage, transfer, and reuse present significant challenges [38], and the TRL of cryogenic propellant on-orbit storage and transfer technology is about 3 to 4; only by overcoming these challenges can the construction of orbital transportation “relay stations” be realized.

As a result, SpaceX, Blue Origin, and European Space Agency (ESA) have already planned relevant development programs for reusable space transfer vehicles, as shown in Table 3, focusing on breakthroughs in key technologies in the near term. In China, the National Natural Science Foundation of China (NSFC) has also supported feasibility and concept studies through major fund projects such as the “On-Orbit Refueling-Based Novel Space Transportation System”. In the future, on-orbit refueling will undoubtedly be a critical ability for long-term and sustainable deep-space activities.

**Table 3.** Cryogenic propulsion-based reusable on-orbit transfer stages under development

System	Propellant	System configuration	Development status and roadmap
Starship spacecraft [72]	LOX/LCH_4_	Six Raptor engines; gross liftoff mass ≈ 1,500 t; 3 vehicle variants: Tanker Starship (Earth–orbit refueling), orbital depot Starship (long-term propellant storage), crewed Starship (lunar landing).	2024: achieved orbital insertion; demonstrated ~10 t intra-vehicle LOX transfer. 2026: planned ship-to-ship on-orbit refueling demonstration; on-orbit testing of HLS Starship; first uncrewed Mars mission planned by year end. 2027: Artemis III crewed lunar landing. 2028: Artemis IV crewed lunar mission.
Blue Moon transfer stage [73]	LOX/LH_2_	Two BE-7 engines; vehicle diameter: 7 m; propellant delivery: 100 t to NRHO/30 t to Mars orbit; active cryogenic cooling (zero boil-off).	2023: full-scale cargo variant unveiled. 2025: development of crewed variant (Mk2). 2026: first lunar landing of cargo variant. 2027: uncrewed flight test of crewed variant. 2029: Artemis V crewed lunar mission; post-mission on-orbit reuse.
InsPoc-2 [74]	LOX	No information	2027: on-ground demonstration 1 [LN2] 2028: on-ground demonstration 2 [LOX and LCH4] 2030: on-orbit demonstration [LOX]

### Long-range aerospace transportation opens up a new direction leading Sci&Tech innovation and industrial development

At present, the cruising speed of mainstream air transportation is less than Mach 1, and intercontinental travel takes up to 12 h. If 1-h global reach can be realized, it will bring a revolutionary leap in the speed and time efficiency of intercontinental travel, and greatly boost global economic, technological, and social progress. With the continuous maturation of reusable launch vehicle technology, transportation costs have been substantially reduced, making such a capability technically and economically feasible [15].

“One-hour global reach” was proposed in 2017 by SpaceX, envisioning the use of reusable rockets or suborbital vehicles to enable ultra-high-speed intercontinental transportation of passengers and cargo. This concept rapidly captured the attention of the defense sector, leading the U.S. to formally initiate the Rocket Cargo program in 2021. This program aims to deliver 30 to 100 t of supplies or personnel to any location on Earth within 1 h, substantially enhancing strategic mobility and rapid response capability, with flight demonstrations planned in the coming years [43–45].

Meanwhile, European countries such as Germany and Italy have proposed similar concepts based on suborbital flight [46]. In China, the China Academy of Launch Vehicle Technology has proposed concepts for utilizing reusable launch vehicles for long-range transportation, completing system-level design studies. In addition, the NSFC has supported conceptual research through the Key Program such as “Overall Design and Control Technologies for Suborbital Long-Range Aerospace Transportation Systems” [47]. These initiatives indicate that space transportation technologies are extending into the domain of terrestrial long-range rapid transport, emerging as a key direction for technological convergence and application exploration.

## Challenges of Large-Scale Space Transportation Systems

In response to the development trends outlined above, future space missions face the following substantial challenges.

### Validation of reusable launch vehicle technologies is incomplete

Although SpaceX’s Falcon 9 first-stage recovery has become routine [35], the “Super Heavy-Starship” system is still working toward full reusability despite tens of flight tests. However, key challenges remain, such as thermal management [48], and the technology readiness is far from sufficient. Table 4 compares the orbital-level reentry environment with that of the first-stage reentry. It can be seen that achieving safe orbital-level reentry and rapid reusability poses a significantly greater challenge.

**Table 4.** Comparison of the orbital-level reentry environment with that of the first-stage reentry

	Maximum speed	Maximum heat flux	High heat flux duration
First-stage reentry	Ma10	Hundreds of kW/m^2^	3 min
Orbital-level reentry	Ma25	About 1 MW/m^2^	≥10 min

Even with SpaceX’s commercially mature first-stage vertical landing technology, the process consumes engine life through multiple re-ignitions and subjects the rocket’s tail section to severe aerothermal loads. This limits the number of reuses and increases turnaround times, resulting in significant losses in orbital payload capacity; thus, it is necessary to further optimize the recovery method and enhance the engine reusable life.

For the rocket’s upper stage, returning from orbit to ground imposes high thermal management requirements and causes even greater payload losses. A feasible alternative is to leave the upper stage in orbit after completing its launch mission and refuel it for next missions.

### Single-mission mass-to-orbit capability is insufficient

The maximum payload capacity achievable in a single launch is a key indicator of the upper performance limit of a space transportation system. It constitutes a fundamental capability underpinning deep-space exploration, cislunar infrastructure construction, and space resource utilization. Moreover, future large-scale on-orbit transfer operations based on on-orbit refueling architectures also rely on sufficient single-launch mass-to-orbit capability.

Historically, the United States and the former Soviet Union successfully developed heavy-lift launch vehicles in the 1960s and 1980s, respectively, each capable of delivering over 100 tons to LEO, thereby providing indispensable support for crewed lunar missions and space station construction. However, supporting future missions aforementioned imposes unprecedented requirements on single-launch payload capacity.

Consequently, the Starship system is redefining single-launch payload capability with an anticipated expendable-mode payload capacity to LEO of up to 250 tons. Russia’s Yenisei heavy-lift launch vehicle was designed to deliver 100 t to LEO in support of crewed lunar and Mars exploration missions, but was suspended in July 2024 by Roscosmos due to funding constraints and high technical complexity.

### Breaking performance limits for space transfer requires fundamental innovations

**Fig. 3.**
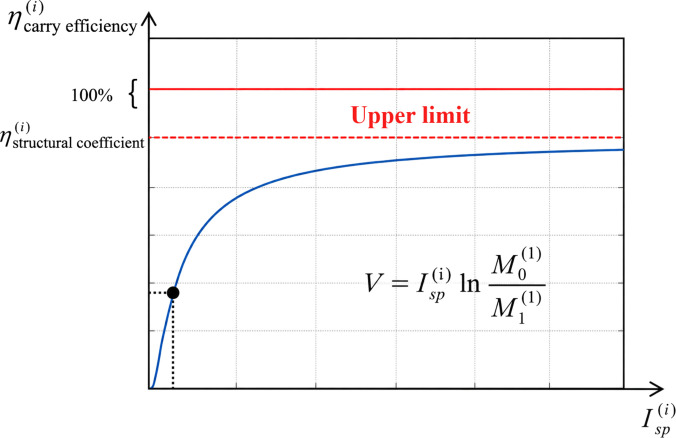
Payload efficiency limitations of launch vehicles under chemical propulsion constraints.

Launch vehicle payload performance is primarily determined by structural mass fraction, engine specific impulse, and liftoff mass, as shown in Fig. 3. These factors are constrained by difficult-to-overcome bottlenecks in a single launch, leaving limited room for improvement and bringing the technology close to both physical and engineering limits from a technical standpoint.

With current chemical propulsion technologies, payload fractions to LEO typically range from 2% to 4%, while high-orbit missions achieve only 0.8% to 1.6%. Equation (2) is a modified form of the Tsiolkovsky rocket equation. It can be seen that when the required velocity increment of the transportation mission remains unchanged, the launch vehicle carrying efficiency is directly correlated with the specific impulse and the rocket structural coefficient. However, the specific impulse of propellants has a physical upper limit, and the rocket structural coefficient is also constrained by the performance limits of structural materials. Consequently, the carrying efficiency of launch vehicles is inherently limited.

$$\begin{array}{c} \eta_{\text{payload fraction}}^{\left(i\right)}=e^{-\frac{\Delta V^{\left(i\right)}}{I_{p}^{\left(i\right)}}}-\eta_{\text{structrue fraction}}^{\left(i\right)} \end{array}$$ (2)

Further improvements would require revolutionary advances in materials, propulsion, and structural design. Meanwhile, liftoff mass cannot be increased indefinitely: As vehicle size and thrust scale up, constraints related to structural strength, manufacturing complexity, transportation and integration, launch site compatibility, and operational safety rapidly intensify, resulting in nonlinear growth in engineering risks and cost. When additional requirements such as reusability, reliability, and rapid turnaround are considered, the feasible upper bound of single-vehicle scale is further reduced. Consequently, breaking current limitations in space access capability necessitates fundamental breakthrough solutions beyond incremental improvements.

As described in the On-orbit refueling enables medium- and high-orbit resource utilization section, on-orbit refueling can effectively enhance the transportation capacity of medium and high orbits, enable the reuse of on-orbit transfer transportation, and reduce the cost of medium-to-high orbit transportation.

### Sustainable cislunar economy lacks scalable economic drivers

**Fig. 4.**
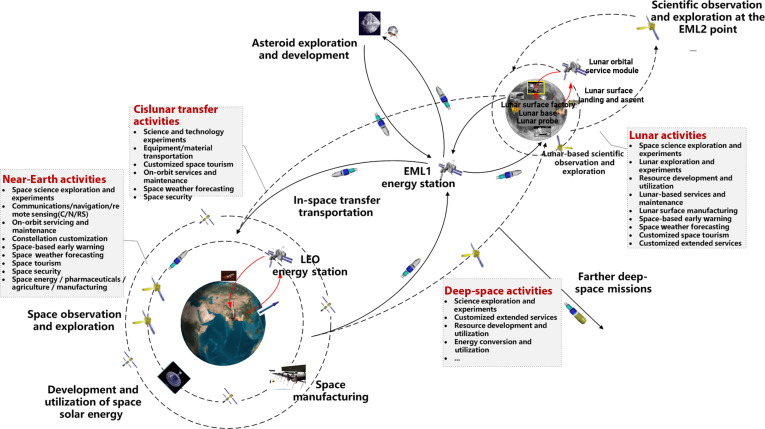
Illustration of economic activities within the cislunar space domain.

The vision of a sustainable cislunar economy is a prerequisite for large-scale space transportation. As illustrated in Fig. 4, both the United States and China have proposed concepts and strategic plans for cislunar economic initiatives, aiming to build space industries covering transportation, resource extraction, energy utilization, and on-orbit services.

However, due to technological constraints and the absence of sustainable commercial business models, these initiatives remain at an early stage. In contrast, as shown in Table 5, SpaceX has successfully established a market-driven space economic model through large-scale LEO mega-constellation deployment. Building on communication services, the company has proposed the concept of “space-based computing power”. This concept extends digital capabilities, including computation and storage, into orbital space. These plans support persistent, large-scale demand for space transportation.

**Table 5.** Space-based computing infrastructure development plans in China and the United States

Program name	Country	Concept and architecture	Status and milestones
Starlink V3 satellite upgrade	United States	Large-scale deployment of space-based AI satellites using Starship; targeting ~100 GW annual data center deployment within 4–5 years	Prototype satellite launch planned for early 2027
Google “Project Sun Catcher”	United States	On-orbit deployment of in-house Trillium-generation TPU chips	Prototype satellite launch planned for early 2027
Zhijiang Laboratory “Trinity” computing constellation	China	Thousand-satellite-scale space computing infrastructure	May 2025: first launch of 12 satellites on a single rocket; target computing capacity: ~1,000 POPS
Space-based data center program [75]	China	Multi-gigawatt space-based data centers in 700–800 km dawn–dusk orbit	2027: breakthroughs in power generation and thermal management; 2030: on-orbit assembly capability; 2035: mass production and on-orbit construction

Future cislunar economic systems are driven by the cost reduction of space transportation, for instance, the reusable Falcon 9 launch vehicle has lowered the historical cost of space access from approximately 10,000 USD/kg to 1,500 USD/kg. This dramatic drop in the cost of accessing LEO has given rise to the large-scale LEO satellite constellation industry and fueled the vigorous development of the low-orbit economy. However, the cost of entering medium and high Earth orbits as well as lunar orbit remains stubbornly high. The cost to medium and high orbits stands at roughly 5,000 to 10,000 USD/kg, while the cost to lunar orbit exceeds 10,000 USD/kg. This has long constrained the industrial development and utilization of medium-high orbits and lunar resources. Therefore, it is urgent to research reusable space transportation technologies for medium-high orbits and deep space, focusing on key technologies such as on-orbit refueling and long-term in-orbit management of cryogenic propellants. Future research will also cover low-cost lunar landing and takeoff technologies, as well as large-scale lunar surface transportation technologies.

## Key Research Directions for Future Space Transportation

**Fig. 5.**
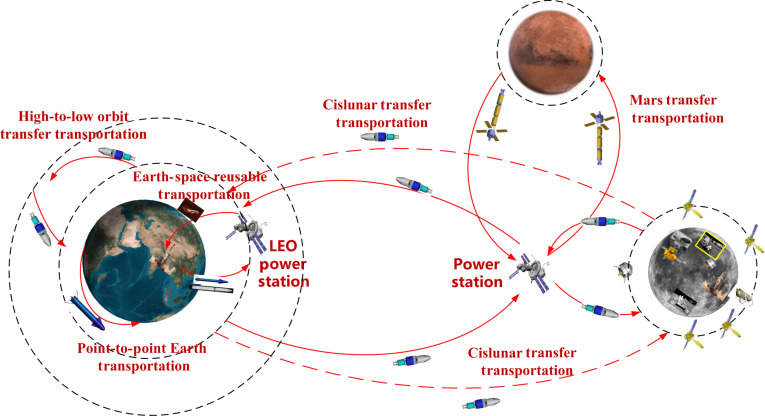
Roadmap for the future development of space transportation systems.

Figure 5 illustrates future space transportation activities that extend across multiple dimensions. The activities span from near-Earth space to cislunar space, deep space, and even the Earth’s surface. This roadmap visualizes the Earth–space round-trip transportation, medium- and high-orbit transfers, Earth–Moon and Earth–Mars shuttles, as well as point-to-point ultra-high-speed terrestrial transport, representing a system-level synthesis of the development trends and key challenges discussed in the preceding sections.

With breakthroughs in reusable launch technologies for near-Earth orbit, the cost and cycle time associated with access to LEO are expected to decrease significantly, enabling “airline-mode” Earth–space transportation as the foundational step. With the growth in single-launch mass-to-orbit capability, large-scale on-orbit transportation capacity is becoming feasible and economical, aided by multiple launches combined with on-orbit refueling. It is anticipated that once routine, large-scale Earth–space and Earth–Moon transportation capabilities mature, space transportation systems will further extend toward deep space, and eventually integrate into everyday human activities to enable high-speed intercontinental travel.

To achieve the above blueprint, priority must be given to addressing the following challenges.

### Active thermal management for hypersonic reentry

To realize the full reusability of launch vehicles and long-range aerospace transportation, it is necessary to achieve the reentry and recovery of high-efficiency and reliable rocket upper stages, where thermal management limits the system development [49]. Unlike first-stage recovery, orbital reentry (exceeding Mach 20) faces extreme aerothermal environments. Heat fluxes reach MW/m^2^, aerodynamic loads exceed 10 tons, and zones above 500 kW/m^2^ span tens to hundreds of square meters. These loads show spatiotemporal nonuniformity with variations exceeding kW/m^2^/s and gradients of hundreds of kW/m. Atmospheric flight duration can exceed 20 min.

Traditional passive thermal protection relies on “blocking” and “resisting” heat. Susceptible to ablation and failure, it may alter aerodynamic shapes and complicates maintenance, failing to meet reliability and reusability needs. The Space Shuttle’s ceramic tiles limited its efficiency, while Starship’s recent tests showed severe surface ablation, underscoring the technology’s persistent challenges.

Both physical mechanisms and flight tests show that passive thermal protection cannot meet future demands for “airline-mode” reusable operations. In contrast, active thermal management uses fluid media to isolate structures and actively regulate heat. Offering superior protection, non-ablative operation, and high reusability, it effectively supports future efficient flight requirements.

Representative challenges include several aspects. First, clarifying the fatigue and failure mechanisms of aerothermal structures under severe and complex coupled mechanical–thermal loading environments is essential. Furthermore, given the significant spatiotemporal variation of heat flux in high-thermal-load regions, it is necessary to address active and adaptive precise heat flux regulation over large areas, as well as the accurate prediction and optimized design of coupled internal–external flow processes. In addition, the scarcity of orbital reentry vehicle sample data limits the extraction of reliable features under small-sample conditions, making it challenging to assess reusable operational reliability.

To address the core technical bottlenecks faced by thermal management for hypersonic reentry, in the direction of adaptive precise heat flux regulation, biomimetic self-pumping self-adaptive phase-change transpiration cooling demonstrates unique advantages: It achieves pump-free self-driving relying on the capillary force of porous media, can automatically match the cooling flow rate according to local heat flux variations, and features non-ablative operation and high reusability. Peixue’s team at Tsinghua University has increased the cooling capacity of this system to over 1.1 MW/m^2^ through fin structure optimization, foam metal insulation, and nanointerface modification [50–54]. In the direction of internal–external flow coupling, cross-scale high-precision calculation methods exhibit critical supporting value. It is necessary to develop a calculation framework for internal–external flow coupling between novel cooling structures and hypersonic environments, solve the problems of excessive computational cost of mesoscopic simulations and empirical parameter dependence of macroscopic simulations, reveal the multi-scale coupling mechanism [55–58], and open up a new path for the mechanism study of cooling structures for multi-physics field-efficient flight vehicles.

### Long-life rocket engine design technologies

Long-life rocket engine design is widely recognized as one of the core enabling technologies for achieving “airline-mode” reuse and low-cost access to space. In addition to meeting high thrust and performance requirements within a single mission, engines must maintain stable performance and structural integrity under multiple start–shutdown cycles and complex flight profiles. Consequently, design objectives are shifting from single-mission reliability toward full life-cycle reliability and predictability.

From an industrial perspective, future reusable launch vehicles are expected to require engines capable of over 100 reuse cycles and cumulative operating times on the order of 10^5^ s, far exceeding the empirical experience base of most existing liquid rocket engines. Even at SpaceX, engine lifetime capability largely relies on extensive ground testing and accumulated flight data; yet, a fully mature, broadly applicable lifetime design and assessment framework has yet to be established.

The long-life rocket engine design is an integrated technology spanning structural mechanics, heat and mass transfer, materials science, reliability engineering, and systems engineering. Major challenges include thermo-mechanical fatigue, creep, and material degradation in thrust chambers subjected to high heat flux and cyclic loading. To address these issues, several design and modeling methodologies have been developed. For thrust chamber life prediction, the sandwich beam theory combined with viscoplasticity constitutive models characterizes the “doghouse” deformation and ligament thinning as continuous temporal functions, enabling computationally efficient service-life prediction [59]. Recent improvements have incorporated fillet geometry effects and optimized thinning formulations accounting for plastic compression [60]. Beyond simplified models, nonlinear finite element approaches employing Chaboche kinematic hardening and Voce isotropic hardening combined with creep formulations have been applied to capture full stress–strain evolution under cyclic thermal loading, with fatigue life evaluated through Smith–Watson–Topper and Wang–Brown multiaxial criteria [61]. Life prediction under combined damage modes typically integrates low cycle fatigue damage via Coffin–Manson, creep damage via Norton model, and ratcheting damage through residual strain accumulation, with total damage assessed by Miner’s linear cumulative rule [62]. Continuum damage mechanics approaches have also been developed to predict ductile rupture of the hot wall ligament [63]. Despite these advances, high-cycle fatigue and reliability risks persist in turbopump components exposed to frequent start–stop and transient conditions, and maintaining combustion stability and control precision becomes increasingly difficult under repeated ignition and deep throttling [64]. Moreover, the lack of robust lifetime prediction models and in-flight health monitoring capabilities introduces uncertainty and risk into reuse operations [65].

### Efficient transfer control and precision sensing of cryogenic propellants

Adopting on-orbit refueling, especially cryogenic propellant on-orbit refueling, can effectively break the payload efficiency limit of launch vehicles and substantially improve the transportation capacity for medium and high orbits. However, efficient transfer control and precise measurement of cryogenic propellants have long been critical challenges, which are essential to ensuring reliable on-orbit refueling.

While storable propellant transfer is mature, cryogenic rapid transfer faces significant challenges in ground and on-orbit scenarios. These arise from rapid flow transitions in complex thermal environments, difficult flow regime identification, and testing limitations [38,39,66], especially for liquid hydrogen.

**Fig. 6.**
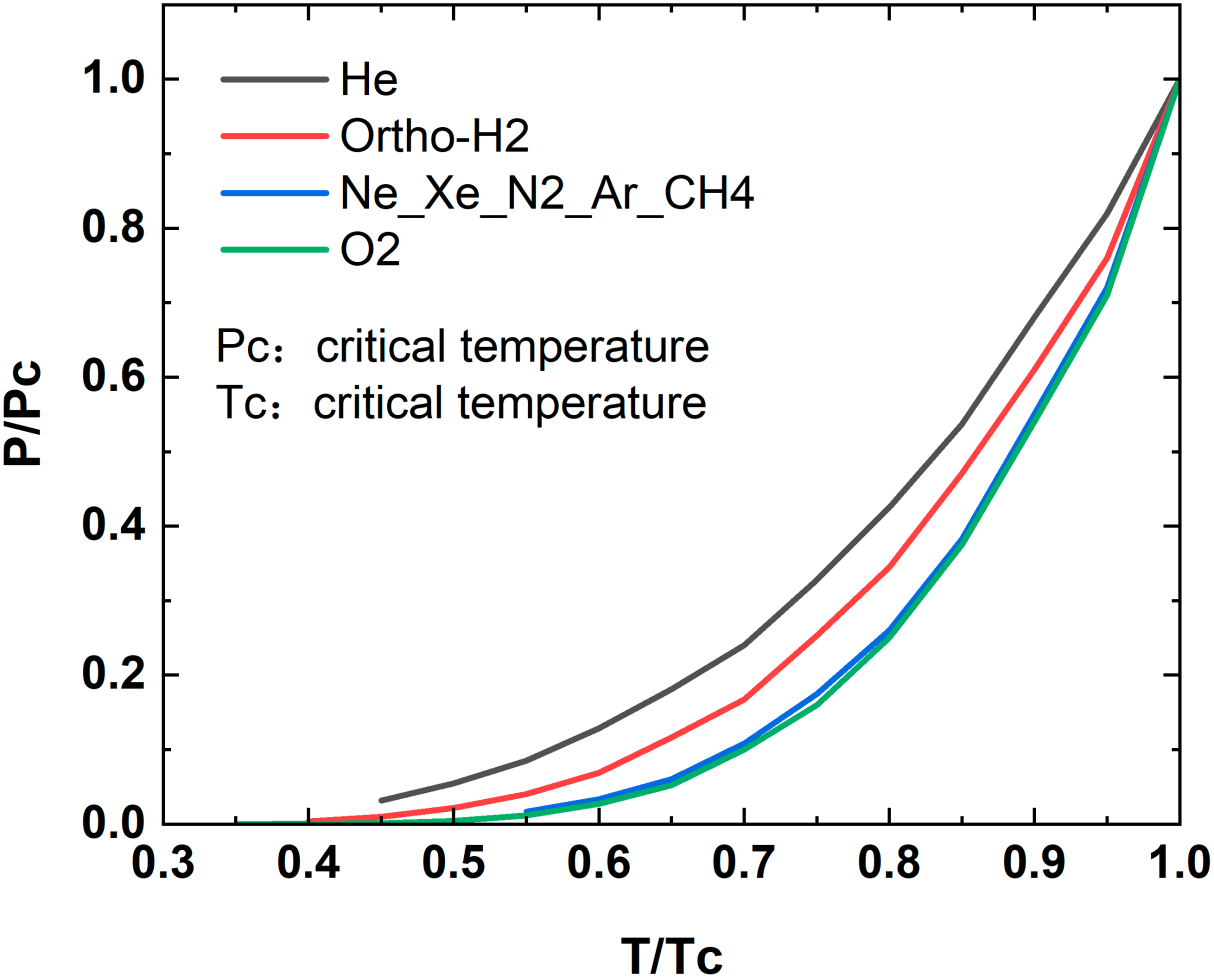
Dimensionless saturated vapor pressure curves of different substances.

Figure 6 presents the dimensionless saturated vapor pressure curves of various substances, showing that, except for helium and hydrogen, most substances collapse onto a single curve, indicating that their thermophysical properties can be derived from corresponding state theory [67]. In contrast, liquid hydrogen and liquid helium display distinct behavior at cryogenic temperatures due to quantum effects and small molecular size, rendering conventional state equations inapplicable.

**Fig. 7.**
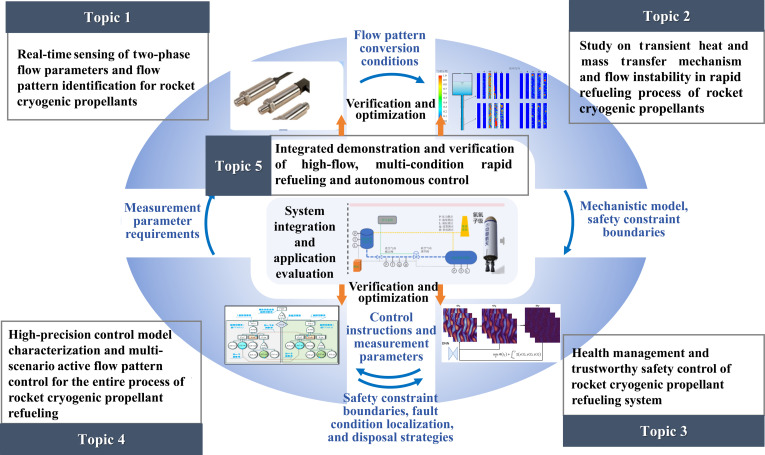
Roadmap of autonomous high-throughput liquid hydrogen transfer control for ground-based operations.

Liquid hydrogen transfer involves vigorous boiling, interface instability, and fluctuating thermodynamics due to complex 2-phase flow behaviors. To support large-flow, multi-condition fueling, fully autonomous control technologies are imperative. These must replace human-in-the-loop procedures, which involve high costs, safety risks, and limited adaptability. Supported by major programs of the NSFC, interdisciplinary approaches are being pursued to address these globally recognized challenges, with representative technical pathways illustrated in Fig. 7.

On-orbit refueling is vital for Earth–space transportation, with cryogenic propellants being the only feasible option for LEO–MEO missions (Table 2). However, microgravity transfer remains challenging; only SpaceX has tested in-vehicle liquid oxygen transfer, while liquid hydrogen refueling is undemonstrated. The extreme low temperature, small molecular weight, and low surface tension of LH₂ make large-flow rapid refueling difficult, leading to issues like high precooling consumption, gas–liquid separation difficulties, challenges in flow identification, measurement errors, and pressure control. Furthermore, spacecraft have minimal structural mass and lack ground-based safeguards. Therefore, investigating microgravity heat transfer mechanisms and developing control methods are essential, alongside technologies for long-term, low-loss on-orbit storage.

### Aero-assisted orbital transfer technologies

**Fig. 8.**
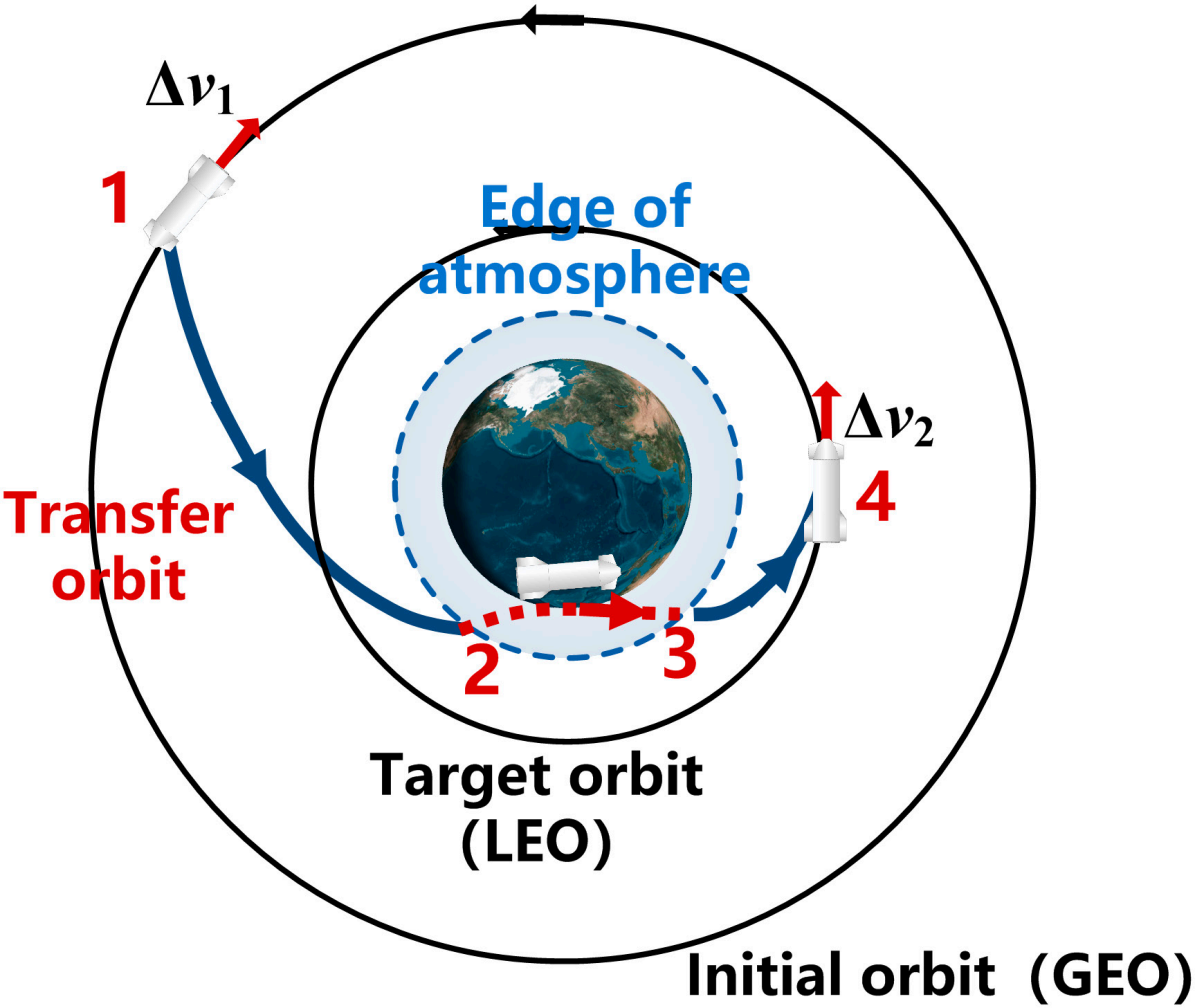
Schematic illustration of aero-assisted orbital transfer maneuvers.

Adopting on-orbit refueling enables the in-orbit reusability of space transfer vehicles, while after completing medium- or high-orbit transfer missions, on-orbit transportation systems must return to LEO. Orbital maneuvers may be performed using propulsive braking or aero-assisted orbital transfer techniques [68–70]. While propulsive braking is technically mature, it incurs substantial propellant consumption. In contrast, aero-assisted maneuvers, as shown in Fig. 8, enable efficient energy dissipation and orbital adjustment within the atmosphere, significantly reducing propellant requirements, decreasing vehicle mass, and lowering overall transportation costs.

Aero-assisted orbital transfer imposes extremely stringent requirements for vehicle aerodynamic configuration and thermal protection systems. In the case of large-scale transfer vehicles, the huge propellant tanks lead to highly constrained aerodynamic shapes that directly influence structural efficiency and payload performance.

Addressing these challenges requires multi-objective integrated optimization of aerodynamic deceleration performance, thermal protection capability, and orbital control accuracy. Furthermore, thermal protection systems must simultaneously achieve lightweight structural design and long-term structural stability under extreme thermal environments. During atmospheric flight, the vehicle must dissipate energy efficiently while ensuring that post-atmospheric orbital parameters meet mission requirements, necessitating coordination between atmospheric trajectories and orbital transfer objectives. In addition, combined aerodynamic and thrust-vector control introduces significant nonlinearities due to fundamentally different actuation mechanisms, posing substantial difficulties for guidance and control.

### Fully electric-driven pressurization and propellant feed control

To realize the reusable operation of space transfer vehicles via on-orbit refueling in the future, reusable missions must be simple and efficient. This requires that after completing one mission, the vehicle does not need replenishment of multiple energy sources in orbit; ideally, only the main propellant needs to be refueled.

**Fig. 9.**
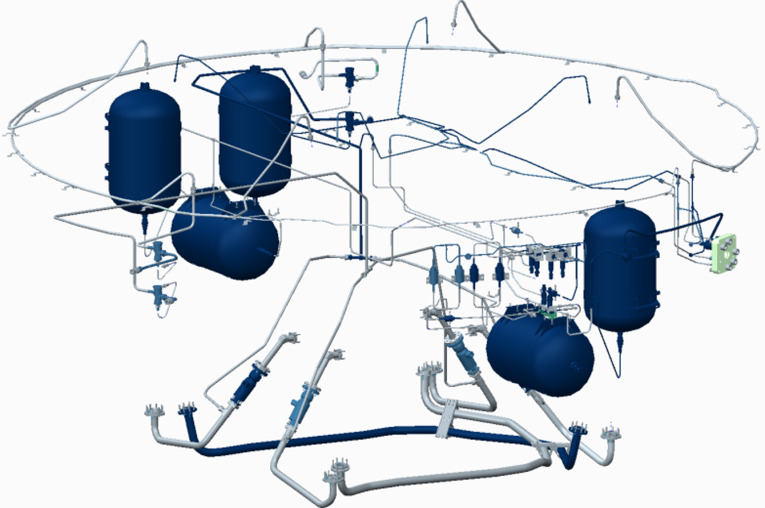
Partial schematic of a representative pressurization and propellant feed system.

Liquid rocket propulsion systems require substantial gas resources and complex pneumatic control networks during flight, primarily for tank pressurization, propellant delivery, and engine operation. Pressurization and feed systems ensure safe and reliable propellant transport to the engine, meeting startup and operational mass flow-rate and pressure requirements while also supporting propellant loading, venting, purging, pre-cooling, and safety functions. As illustrated in Fig. 9, traditional pneumatic architectures involve complex piping, high-pressure gas sources, and intricate control logic. Engine startup typically relies on gas-driven turbopump spin-up, requiring propellant dumping for thermal conditioning and large onboard gas bottles to maintain tank pressurization and inlet pressure margins.

Compared with pneumatic control, electrically driven architectures offer reduced structural complexity and improved reliability, representing one of the most effective approaches for enhancing performance through simplified design. Electrically pumped liquid rocket engines, first proposed by Chazen in 1984 [71], utilize high-energy-density batteries and electric motors to drive propellant pumps, eliminating high-temperature components such as turbines and gas generators. This configuration reduces the mass of pressurant gas systems and allows all propellant to be used for thrust generation, thereby improving effective specific impulse. Such systems are particularly well suited for advanced reusable on-orbit transportation system.

For fully reusable on-orbit transfer systems, minimizing the amount of consumables requiring on-orbit replenishment—such as pressurant gases—is essential, because onboard gas resupply complicates long-duration orbital operations and requires large gas storage capacity. In contrast, electrically driven systems can exploit solar power generation, offering clear advantages in energy sustainability and long-term operability. However, electric drive cannot completely eliminate the need for pressurant gases, making the development of regenerable in situ pressurant production technologies (e.g., helium or nitrogen generation) equally important.

For electric-driven pressurization and feed systems, it is necessary to elucidate the evolution of mechanical and electromagnetic properties of motor materials across wide temperature ranges: to develop lightweight, low-power, deep-cryogenic electric motors and integrate them with large-diameter, deep-cryogenic, high-pressure electrically actuated valves, including advanced sealing and thermal management solutions. For electric-driven engine startup, it is essential to uncover the flow, heat transfer, and phase-change mechanisms governing electric-pump pressurization under ultralow temperature and microgravity conditions, to understand the impact of complex 2-phase flows on pump performance, and to achieve stable thermal–mass transfer control, wide-range continuous throttling, and reliable operation of cryogenic electric pumps under extreme environments.

### Integrated aerodynamic–control design for high lift-to-drag ratio and high payload fraction vehicles

**Fig. 10.**
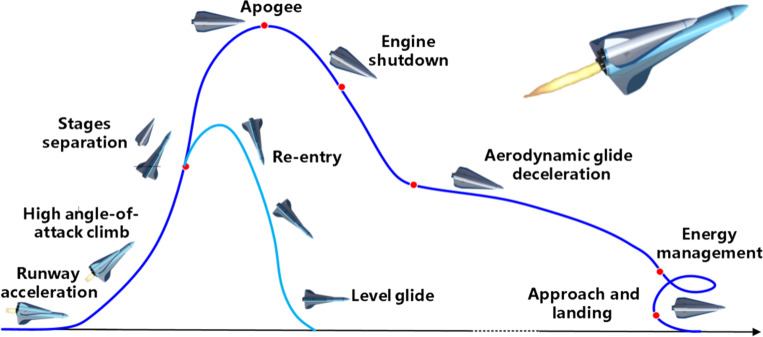
Representative flight profile of point-to-point ultra-high-speed terrestrial transportation.

The evolution of reusable launch vehicle technology enables capabilities for long-range logistics, rapid material resupply, and 1-h intercontinental travel. Notably, emerging point-to-point ultra-high-speed transportation systems, illustrated in Fig. 10, represent a critical application. To achieve global surface coverage and substantially enhance transportation efficiency, such systems must satisfy requirements for high payload capacity, long range, and precise controllability [46,47].

However, inherent physical trade-offs exist among aerodynamic performance, payload volume, thermal protection, and flight control. Pursuing a high lift-to-drag ratio to reduce energy consumption and enable long-range gliding often comes at the expense of internal volume, whereas increasing payload packing density tends to significantly degrade lift-to-drag ratio and increase energy demand. Under hypersonic flight conditions, pitch, yaw, and roll motions are strongly coupled. Changes in one channel can affect aerodynamic and dynamic characteristics in the others. As a result, traditional decoupled control strategies become ineffective, significantly increasing the risk of mission failure. Consequently, achieving a 1-h continental travel requires breakthroughs in aerodynamic–control coupled optimization, accurate prediction and robust stabilization of flight states during large-angle high-speed gliding maneuvers, as well as integrated guidance and control design for precision landing under high-dynamic and strongly constrained conditions.

## Conclusions

The large-scale space transportation represents the next major expansion domain beyond first-stage recovery and reuse. It aims for the “dual-hundredfold” goal, with a hundredfold increase in both launch mass and frequency, requiring significant advances through interdisciplinary advantages to dramatically enhance capabilities. At the same time, it will integrate the achievements of heavy-lift launch vehicle and first-stage recovery technologies, and, building on these foundations, transition into the era of “airline-mode” flight, greatly reducing the cost of space travel. This system is a crucial capability for humanity to transcend Earth’s constraints, develop the space economy, and build the cislunar econosphere. As fully reusable technologies continue to mature, space transportation systems are poised to reshape the current intercontinental travel ecosystem. Global 1-h high-speed travel emerges as a new mode of transportation.

To achieve the aforementioned vision, many advancements are required on the current technological basis, including achieving full reusability of launch vehicles, significantly increasing the single-launch payload capacity, substantially enhancing space transportation capability, and greatly reducing the cost of cryogenic propellant transfer, storage, and on-orbit refueling.

The critical transition of large-scale space transportation systems hinges on overcoming these interrelated capability bottlenecks, which constitute the central focus of this paper. These key technologies are simultaneously distinct, interdependent, and highly synergistic. Breakthroughs in these areas are expected to catalyze the emergence of new industries, drive explosive growth in space-related economic activities, and fundamentally reshape the global landscape of technological competition and strategic balance.

As such, large-scale space transportation should not be regarded merely as an incremental enhancement of launch capability, but rather as a systemic transformation of how humanity accesses, utilizes, and economically integrates space.

## Data Availability

All data needed to evaluate the conclusions in the paper are present in the paper and/or the Supplementary Materials.
